# Hookworm Infection among Pregnant Women at First Antenatal Visit in Lira, Uganda: A Cross-Sectional Study

**DOI:** 10.1155/2020/8053939

**Published:** 2020-06-26

**Authors:** Felister Apili, Stephen Ochaya, Charles Peter Osingada, Scovia Nalugo Mbalinda, David Mukunya, Grace Ndeezi, James K. Tumwine

**Affiliations:** ^1^Department of Nursing, College of Health Sciences, Makerere University, Kampala, Uganda; ^2^Faculty of Health Sciences, Lira University, Lira, Uganda; ^3^Department of Clinical Pathology, Academic Hospital, Uppsala, Sweden; ^4^Department of Immunology and Microbiology, Gulu University, Gulu, Uganda; ^5^Center for Intervention Science in Maternal and Child Health, University of Bergen, Bergen, Norway; ^6^Department of Paediatrics and Child Health, College of Health Sciences, Makerere University, Kampala, Uganda

## Abstract

**Background:**

Hookworm infection in expectant mothers has adverse health effects on both the mothers and their unborn babies. Foetal effects are known to include intrauterine growth retardation and physical and mental growth retardation, while the mothers may develop anemia which could potentially result in death. Unfortunately, little is known about factors that may predispose a pregnant woman to infection by hookworm. In this study, we strived to determine not only the prevalence of hookworm infection among pregnant women attending their first antenatal visit during the current pregnancy in a local health center in northern Uganda but also factors that might predispose them to hookworm infection.

**Method:**

This cross-sectional study was conducted among 346 pregnant women from Ogur Health Center IV located in Lira district, northern Uganda. Stool samples were collected from each study participant and analyzed for hookworms. The independent variables listed in this study (participant's sociodemographic characteristics, preconception care, and sanitation factors) were obtained using a structured questionnaire. Data analysis, including calculation of adjusted ratios, was performed using STATA software (version 14).

**Results:**

Prevalence of hookworm infection among pregnant women who attended their first antenatal visit at Ogur Health Center IV was 11% (*n* = 38). After controlling for confounders, factors found to be significantly associated with this infection among pregnant women here were gardening barefooted (adjusted odds ratio (AOR), 3.4; 95% confidence interval (CI), 1.6 to 7.5; *P* < 0.001) and fetching unsafe water shared with animals for domestic uses (AOR, 2.8; 95% CI, 1.3 to 6.2; *P* value of 0.002).

**Conclusion:**

Hookworm infection among pregnant women at Ogur Health Center IV in Lira district, at 11%, is a public health concern and significantly associated with barefoot gardening and fetching water from unsafe sources shared with animals. We, therefore, recommend that special emphasis during routine prenatal health education be placed on the use of protective footwear during farming and fetching water for domestic use from protected safe sources. *Author Summary*. Hookworm infection is a parasitic condition that more often goes unnoticed, yet it presents immense detrimental effects, especially to pregnant women and their unborn children. It is a chronic disease with accruing effects of blood depletion resulting in anemia. Anemia is, by far, one of the major causes of maternal morbidity and mortality in Uganda. Pregnant women are more prone to hookworm infection by virtue of their compromised immunity, secondary to the physiological process of pregnancy. We demonstrated here that hookworm infection still exists among pregnant women in Uganda. We also showed that gardening barefooted and fetching water for domestic uses from unsafe sources shared with animals were major factors associated with this helminthic infection. This study provides evidence necessary to influence decision making on prevention of hookworm infection in the study area.

## 1. Background

Hookworm infection is a major public health concern. The two known species of concern are *Ancylostoma duodenale* (*A. duodenale*, causes ancyltomiasis) and *Necator americanus* (*N*. *americanus*, causes necatoriasis) [1]. These species have similar lifecycles and are difficult to differentiate by the traditional light microscopy. The larval stages require unique but sometimes overlapping environments, mostly soil, water, and temperature for sustenance. *A*. *duodenale* was found mostly in the Middle East, North Africa, India, and Southern Europe before it was eradicated. In contrast, *N*. *americanus* is found in America (tropical and semitropical regions), Sub-Sahara Africa, Southeast Asia, China, and Indonesia. Both species of hookworm infect primarily by way of the mature infective third-stage larva penetrating human skin [2] and through oral ingestion in case of *A*. *duodenale* [3]. Children and pregnant women are particularly vulnerable to hookworm infection [4]. Hookworm infection during pregnancy is associated with adverse health effects in expectant women and the unborn children [5]. Hookworm, a blood-feeding parasite that lives in the small intestine, is common in tropical countries largely due to a favourable climate in those countries [6], and in poor countries due to socioeconomic factors such as low quality of water for domestic use fetched from sources shared with animals, lack of, or poor utilisation of pitlatrine and poor personal hygiene in general [7]. This infection is characterized by ground itch at the point of entry, fever, nagging cough, fluctuating appetite, weight loss, and diarrhoea [8].

Hookworm infection has been reported to predispose victims to anemia [9], and in Uganda, hookworm infection is one of the few common factors that cause anemia; a disease that is among the top 10 causes of maternal morbidity and mortality [3]. Anemia is also one of the top five causes of hospital-based mortality in women and children [3] and is generally associated with poor birth outcomes like preterm labor, low birthweight [10], intrauterine growth retardation, extrauterine growth retardation, delayed sexual development, and early infant death [11, 12]. The prevalence of anemia among pregnant women in Uganda is estimated to be 32% [13]; 20.2% of which is attributable to hookworm infection [14]. Because of this, the Uganda ministry of health adopted the WHO recommendation of routine biannual deworming for girls and women of reproductive age and twice in pregnancy during the second and third trimesters [15].

Factors that may predispose an individual to hookworm infections are demographics, social, economic, and biological status. Socioeconomic status of families greatly influences the quality of life [16]. Poor communities often fail to access basic sanitation facilities, walk barefooted, and use farm tools with bare hands, all of which escalate the risk of hookworm infection [17]. Farming, the main economic activity in rural areas, keeps the rural population in constant contact with infected soil [12].

In Uganda, the prevalence of hookworm infection as reported from previous studies was relatively similar across different geographical locations. For instance, 45% of Entebbe population are infected [18], 40.5% in Mayuge, and 51% in Tororo. In Ethiopia, it is reported to be 36.1% [19]. Unfortunately, very few published studies have been done specifically among pregnant women in these and other geographical areas. We believe this to be an oversight because preventing hookworm infection improves pregnancy outcomes since the unborn is always the most affected in this scenario. It was, therefore, imperative to study the prevalence of hookworm infection among pregnant women and identify the factors involved. In this study, we have taken a first step in that direction in northern Uganda with pregnant women attending their first antenatal visit at Ogur Health Center IV in Lira district. The goal was to assess the prevalence of hookworm infection and identify preliminarily factors that predispose a pregnant woman in this area of Uganda to this parasitic infection.

## 2. Methods

### 2.1. Study Design

This was a cross-sectional study conducted between December 2017 and February 2018 among pregnant women attending their first antenatal visit (for that particular pregnancy regardless of the gestational age at the time of contact). Only pregnant women who consented to participate in the study and had not received routine mebendazole treatment during the current pregnancy were enrolled. Also, excluded were severely ill pregnant women who could not disclose personal information or bring stool samples. Similarly, those who met the criteria, consented to participate but failed to provide stool samples were excluded as well. Each participant provided approximately 10 g of valid stool sample uncontaminated with water or urine. No visual evidence of water or urine in a stool sample assured validity.

### 2.2. Study Area

The study was done at Ogur Health Center IV, a rural site located approximately 30 km from Lira town (Lira district, northern Uganda) along Kitgum road. The health center is a mid-level health facility that offers basic and emergency obstetric services and operates an antenatal clinic every weekday except public holidays. Pregnant women attending their first antenatal care (ANC) are booked on all those days. Although it has only 24 beds, the health center offers general health care services for a catchment area of 106 villages with about 40,269 people according to Lira district population statistics of 2018. On average, the monthly outpatient attendance is about 12,000 patients of which 2,600 are pregnant women seeking antenatal care. We chose to study this rural site because of the suitable environment and practices that favor hookworm proliferation. For example, land cultivation which is the major economic activity among the rural population [20] provides a favourable climate alongside contact with infected soil on bare hands and feet during cultivation for hookworm infection [21]. Additionally, Lira district, the location of Ogur Health Center IV, is currently recovering from a 20-year civil war and is extremely poor. Former war refugees have since relocated to villages at the end of the war where they live in semipermanent houses made of mud blocks without concrete floors. They often share water from unprotected wells with animals, more so, during the dry seasons when water is scarce. The latter scenario further exposes the rural population to hookworm infection [22].

### 2.3. Sample Size Determination

The sample size for this cross-sectional study was calculated using the Kish Leslie formula [23]: *n* = *z*^2^*pq*/*d*^2^, where *n* is the minimum sample size, *z* is the standard score corresponding to 5% level of significance (i.e., 0.05 and *z* = 1.96), *p* is 1% (the prevalence of hookworm infection among pregnant women), *q* is 99%, and *d* is 5% confidence limit (the proportion of sampling error). We assumed that the prevalence of hookworm infection among pregnant women in Ogur was similar to the 45% obtained from a randomized controlled trial done in Entebbe, Uganda [18]. With an alpha value of 5% and a precision value of 5%, this study needed 380 participants.

### 2.4. Sampling Method

To attain the required sample size within the study timeframe, consecutive sampling was used to recruit participants as follows. A team was stationed at the antenatal clinic who interviewed women as they arrived before the antenatal services commenced. Their arrival usually began at around 10 to11 am local time (the late start time is to give the same staff shared between the delivery ward and antenatal clinic at the health facility ample time to complete the latter). Interviewing was continued daily (except on public holidays and weekends) until the targeted sample size was achieved. A comprehensive interviewer-administered questionnaire was developed in English language and translated into Lango, the native language spoken in the study area. For applicability and accuracy and to find out how clearly respondents understood and responded to questions as intended, the questionnaire was pretested one week prior to data collection. Broadly, the questionnaire was used to gather data on social demographic factors, obstetric characteristics, and environmental-related factors. Six items in the questionnaire (presented in Table 1) measured sociodemographic characteristics and ten items measured preconception factors. Prevalence was derived from microscopic stool analysis where the number of all positive slides for ova, larva, or adult hookworm were summed up and divided by the respondent sample size. The results are expressed as percentages in this study.

### 2.5. Study Variables

#### 2.5.1. Dependent Variables

The dependent variable in this study was hookworm infection among pregnant women at their first ANC visit in Ogur Health Center IV. Stool samples were examined for hookworms using direct and formol-ether-based light microscopic methods. The presence of ova, larva, or adult hookworms indicated hookworm infection; the absence meant no hookworm infection.

#### 2.5.2. Independent Variables

The independent variables were sociodemographic characteristics of the pregnant women, preconception care, and sanitation factors. Age was categorized in years as ≤20, 21-30, and>30; a participant's religion was considered to be either Christian or Moslem; marital status was taken to be married or single; education status as either no formal education, primary, or secondary or higher; a person was considered a resident if she lived in this community or visited for a month or more. Occupation was civil servant and/or businesswoman (paying employment), housewife, and/or peasant farmer (nonpaying employment). Proper pit latrine utilisation was considered if one reported disposing all faecal materials of both children and adults into the pit latrine. Boreholes, protected spring wells, and taps were taken as safe sources of domestic water supply while unprotected spring wells were considered unsafe water sources. These variables were measured using a pretested twenty-one-item tool developed in-house.

### 2.6. Participant Recruitment and Data Collection Procedure

Each participant was required to provide a stool sample before she could respond to the questionnaire. All 380 stool sample containers used in this study were paired up and precoded similarly with corresponding questionnaires to avoid mixing up of samples. Participants were educated about avoiding sample contamination with urine or water during collection; they provided freshly voided stool samples. The samples were collected from a cleaned toilet provided with toilet paper and hand washing facility at the health center. Even then, 34 participants failed to empty their bowel hence could not proceed to answer the questionnaires. Stool analysis was done within 30 min of collection. The interviewer-administered questionnaires were used to gather information about sociodemographic and potential factors associated with hookworm infection (see Sampling Method). The interviewer checked all questions immediately after questionnaire completion for completeness before disengaging with a participant and, where necessary, sought clarifications. Laboratory results were disclosed to participants on the same day of testing and before they left the health center. A brief health talk was conducted pertaining to hookworm infection in pregnancy and measures of preventing exposure including proper use of pit latrine for both children and adults, building protected water sources, and wearing hand and foot protective gears during farming to avoid contact with hookworm-infected soil.

### 2.7. Specimen Processing and Analysis

Stool analysis was done at the Ogur Health Center IV laboratory by two qualified and experienced laboratory technicians. Semisolid samples were examined within 30 minutes of collection, while the rest were examined within an hour of collection without refrigeration to prevent destruction of the morphology of ova and adult warms.

Two methods of stool analysis were used simultaneously. Fresh warm stool was analyzed by direct microscopy at two different magnifying powers (10x and 40x) to observe eggs, larva, and adult hookworms. Some portions of the samples were examined by light microscopy after performing formol-ether concentration. Although direct microscopy is cheap and easy to perform under our field conditions, it is only sensitive to heavy infections. Formol-ether concentration method for intestinal parasites, on the other hand, is more sensitive at low hookworm infection and therefore was used to minimize false negatives. As such, all samples which qualified as negative for hookworms by direct microscopy were also subjected to the formol-ether concentration method.

### 2.8. Statistical Analysis

A statistical software package STATA 14.0 (Stata Corp, College Station, TX, USA) was used to summarize the data we collected in tables. Continuous descriptive variables were presented as means and standard deviations. Categorical variables were presented as proportions. We performed bivariable and multivariable logistic regressions to determine the association between independent factors and hookworm infection. Factors known from the literature to be predictors of hookworm infection and those with a bivariable *P* value < 0.25 (as long as they were not in the casual pathway and they were not strongly collinear with other independent variables) were considered for the initial multivariable model [1]. To assess for collinearity, factors were considered strongly collinear if their variance inflation factor was greater than 10. In case of collinearity, the factor with a stronger measure of association with the outcome variable was retained and the other dropped. We generated the final model using the method of purposeful selection of variables as described by Hosmer and Lemeshow [24]. A variable was called a confounder if it changed the unadjusted measure of association by 10% or more. The final model included all confounders and was tested for goodness-of-fit using the Hosmer and Lemeshow goodness-of-fit test [24].

### 2.9. Ethics

The Research and Ethics Committee of Makerere University School of Health Sciences approved this study (reference number #SHSREC REF: 2017-060). Prior to enrolment, written informed consent was obtained from each participant, clearly explaining the study processes, benefits, discomforts, and their rights to refuse or withdraw from the study at any time without consequences. Confidentiality and privacy were maintained throughout the study, and no direct identifier was captured on questionnaires. Pregnant women under the age of 18 years were regarded as emancipated minors who could consent [25]. Declining to provide stool samples or to respond to questions asked did not in any way obscure access to routine ANC. Results were provided to the participants. All individuals with positive results were helped to access treatment. Education about preventing hookworm infection was provided to every participant.

## 3. Results

### 3.1. Sociodemographic Characteristics of Participants

We interviewed 346 participants ranging in age from 15 to 42 years (mean 23 ± 6 SD). Most participants were Christians, married, had less than 7 years of formal schooling, and were mainly peasant farmers and/or housewives. The overall social demographic characteristics of the participants are summarized in Table 1.

### 3.2. Prevalence of Hookworm Infection

This was obtained from the summation of the number of positive results by both direct microscopy and formol-ether methods then expressed as a percentage of the response sample size (346) as is shown in Table 2. Eighteen out the 346 tested samples were found positive for hookworm infection using direct microscopy. Eleven of which were quantified as light infection whereas seven were heavy hookworm infection.

### 3.3. Distribution of Hookworm Infection

Hookworm infection was more common among resident, young, married, Christian pregnant women, with less than seven years of formal education, and peasant farmers. This helminthic infection was also predominant among women who reported to have given birth before and currently in their first trimester of pregnancy and fetched domestic water from unsafe sources shared with animals. The infection was similarly higher among participants who practiced open defecation. The results are presented in Table 3.

### 3.4. Factors Associated with Hookworm Infection

Results of both bivariable and multivariable analyses of the data are presented in Table 4. During bivariable analysis, all factors apart from marital status were significant (*P* values < 0.05). The multivariable analysis, however, revealed only two statistically significant factors: gardening barefooted and fetching domestic water from sources shared with animals. These results are presented in Table 4.

## 4. Discussion

This study finds that hookworm infection is prevalent among pregnant women at their first ANC visit during the current pregnancy in Ogur Health Center IV. Women who reported to have given birth before were found to have a higher prevalence (71.1%, *n* = 38) than those pregnant for the first time. Several potential factors known to be associated with hookworm infection were confirmed as confounders. Gardening barefooted and fetching unsafe water from sources shared with animals were strongly associated with hookworm infection in this population of pregnant women.

This finding that hookworm infection is prevalent in a rural community of Uganda confirms the notion that this helminthic parasite is predominate in tropical areas [6] owing to conducive environmental conditions for sustenance. It also confirms that the proximity to hookworm-infected soil is a major health concern for pregnant women. The 11% prevalence of hookworm infection among pregnant women as at their first ANC visit found here was lower than those obtained from other studies in Uganda. This is not surprising since this study was conducted during the dry season in northern Uganda (December 2017 and February 2018) when no land cultivation was being done, open wells were mostly dry and temperature was probably too high for hookworms to survive in the soil. We believe this thesis fits here because studies elsewhere have demonstrated seasonal discrepancy. In Bangladesh, for example, a low prevalence of hookworm infection was found during the winter season (19.4%) when compared to the rainy season (29.3%) [26]. In India, a higher prevalence of hookworm infection (80.5%) was reported in autumn than the 43.9% in spring [27].

The prevalence of hookworm infection as determined in this study is similar to those observed in Kenya (3.9%) [28] and Ghana (13.9%) [29]. This could probably be attributed to the routine deworming practice within this vulnerable group. Locally, the prevalence observed here is much lower than the 45% obtained in a double-blinded randomized controlled trial among a similar study group in Entebbe, Uganda [18]. The discrepancy could have resulted from the fact that Ndibazza et al. [18] had a big sample size of 2,507 participants and conducted their study all year round for nearly two years and among a fishing community living around the shores of Lake Victoria where majority of the people do not have proper sanitation facilities and the area has a weather conducive to hookworm proliferation [30]. In other studies, the prevalence of hookworm infection in two communities in Uganda was 40.5% in Mayuge [14] and 51.6% in Tororo located in eastern Uganda [1]. These studies were, however, conducted among the general population and not pregnant women; which explains the difference in results in relation to this study findings. Additional studies from two other Sub-Saharan countries involving large sample sizes found that the prevalence of hookworm infection in Ethiopia was 36.1% [19] and among pregnant women in Ghana was 13.9% [29]. The latter is similar to the prevalence of hookworm infection among pregnant women at their first ANC visit in Ogur.

### 4.1. Factors Associated with Hookworm Infection

This study has shown that gardening barefooted (AOR, 3.4; 95% CI, 1.6 to 7.5; *P* < 0.001) and fetching water for domestic uses from unsafe sources shared with animals (AOR, 2.8; 95% CI, 1.3 to 6.2; *P* = 0.002) were strongly associated with hookworm infection among pregnant women attending first antenatal visit in Ogur Health Center IV as is seen in Table 4. The association between gardening barefoot and infection with hookworm emanates from the fact that rural women do many fieldwork including harvesting and weeding crops without protective footwear, hence exposing them to infective hookworm larva thereby increasing risk of getting infected. In addition, most women in rural northern Uganda, from personal observation, are often barefooted. Barefooting was reported to result from the social norms of not wearing them while in the garden or when at home generally and from the inability to afford shoes. Again, from personal observation and experience, shoes in the study area are spared for special occasions like church and marriage functions.

Association between gardening barefooted, fetching water for domestic uses from unsafe sources shared with animals, and hookworm infection demonstrated in this study is consistent with the life cycle of the more prevalent *N*. *Americanus* [3]. Preventative measures should therefore target curbing these factors to break the cycle of hookworm infection. A cohort study conducted in Thailand among the rural population reported associations between barefooting or raising buffalos near the house and hookworm infection [31], a result very similar to our finding. Similar findings were also obtained in a study from Ethiopia in which hookworm infection was demonstrated to be significantly associated with walking barefooted as well [19]

In 2014, Strunz and colleagues conducted a meta-analysis and systematic review of 94 studies to determine the association of improved water, sanitation, hygiene (WASH) and soil-transmitted infections including hookworm. Five of those studies were randomized controlled trials and the rest cross-sectional studies. Their review demonstrated a significant association between wearing shoes and decreased odds of hookworm infection [22]. However, a recent cross-sectional study in Ethiopia among 283 elementary and secondary school students failed to demonstrate any significant associations between hookworm infection and sources of domestic water or shoe wearing [7] contrary to our study findings.

Open-ground defecation and poor utilisation of the pit latrine were some of the factors independently found to be associated with hookworm infection in this study. This was probably because the study site is rural which increases risk of hookworm infection when the occupants choose to go to the bush to help themselves, without putting on shoes. Secondly, barefooting and improper use of latrines by defecating on the floors can contribute to hookworm infection. However, these variables were ruled out as confounding factors during the multivariable data analysis process of this study.

## 5. Conclusion

We have shown in this study that hookworm infection during pregnancy in Ogur, at a prevalence of 11%, is still a public health concern that is associated with gardening barefooted and fetching water for domestic uses from unsafe sources shared with animals. This is notwithstanding our main limitation of low predictive power here to study factors associated with hookworm infection. In the interim, we encourage income-generating activities as part of the routine prenatal care education so that pregnant women can afford protective footwear and access to safe water in order to prevent hookworm infection.

## Figures and Tables

**Table 1:** Sociodemographic characteristics of pregnant women attending their first ANC at Ogur Health Center IV (*n* = 346).

Characteristic	Frequency	Percentage (%)
Age (years)		
≤20	171	49.4
21 to 30	138	39.9
>30	37	10.7
Religion		
Christian	333	96.2
Moslem	13	3.8
Marital status		
Married	302	87.3
Single	44	12.7
Education level		
None	299	86.4
Primary	34	9.8
Secondary or higher	13	3.8
Resident		
Yes	328	94.8
No	18	5.2
Occupation		
Unpaying employment	279	80.6
Paying employment	67	19.4

**Table 2:** Results of stool sample analysis of pregnant women attending their first ANC at Ogur Health Center IV, Lira district, northern Uganda.

Method	Stool sample (*n* = 346)	
	Positive	Negative
Direct microscopy	(18)	328
Light infection (<1,000 ova/larva/adult hookworms)	11	—
Heavy infection (>1,000 ova/larva/adult hookworms)	7	—
Formol-ether concentration	(20)	308
Light infection (<1,000 ova/larva/adult hookworms),	19	—
Heavy infection (>1,000 ova/larva/adult hookworms)	1	—

**Table 3:** Distribution of hookworm infection among pregnant women attending first antenatal visit at Ogur Health Center IV, Lira district, northern Uganda.

Variable	Hookworm positive (*n* = 38)	Hookworm negative (*n* = 308)
Age (years)	*n* (%)	*n* (%)
≤20	17 (44.7)	154 (50)
21-30	14 (36.8)	124 (40.3)
>30	7 (18.4)	30 (9.7)
Religion	*n* (%)	*n* (%)
Christian	36 (94.7)	297 (96.4)
Moslem	2 (5.3)	11 (3.6)
Marital status	*n* (%)	*n* (%)
Single	9 (23.7)	35 (11.4)
Married	29 (76.3)	273 (88.6)
Education	*n* (%)	*n* (%)
None	11 (29.0)	46 (14.9)
Primary	26 (68.4)	218 (70.8)
Secondary or higher	1 (2.6)	44 (14.3)
Occupation	*n* (%)	*n* (%)
Peasant farmer/HW^a^	33 (86.8)	248 (80.5)
Civil servant/BW^b^	5 (13.2)	60 (19.5)
Resident	*n* (%)	*n* (%)
Yes	38 (100)	291 (94.5)
No	0 (0)	17 (5.5)
Gestational age	*n* (%)	*n* (%)
1st trimester	20 (52.6)	154 (50.0)
2^nd^-3^rd^ trimester	18 (47.4)	154 (50.0)
Gravidity	*n* (%)	*n* (%)
Primegravida	11 (28.9)	70 (22.7)
Multigravida	27 (71.1)	238 (77.3)
Water source	*n* (%)	*n* (%)
Unsafe	14 (36.8)	47 (15.3)
Safe	24 (63.2)	261 (84.7)
Boiling drinking water	*n* (%)	*n* (%)
Yes	2 (5.3)	58 (18.8)
No	36 (94.7)	250 (81.2)
Pit latrine utilisation	*n* (%)	*n* (%)
Yes	6 (15.8)	14 (4.6)
No	32 (84.2)	294 (95.5)
Gardening shoes	*n* (%)	*n* (%)
Yes	10 (26.3)	121 (39.3)
Never	28 (73.7)	187 (60.7)
Floor type	*n* (%)	*n* (%)
Concrete	3 (7.9)	58 (18.8)
Earth	35(92.1)	250 (81.2)

^a^Housewife. ^b^Businesswoman.

**Table 4:** Factors associated with hookworm infection among pregnant women attending their first ANC visit at Ogur Health Center, Lira district, northern Uganda.

Variable	Crude OR^a^ (95% CI)	*P* value	Adjusted OR^a^ (95% CI)
Mother's age			
≤20	1		—
21-30	1.0	0.953
>30	2.1	0.128
Religion			
Moslem	1		—
Christian	0.67	0.607
Marital status			
Single	1	1	—
Married	0.41 (0.18-0.94)	0.036	
Mother's education			
None	1		1
Primary	0.50 (0.23-1.08)	0.078	0.70 (0.30-1.6)
Secondary or higher	0.10 (0.01-0.77)	0.027	0.17 (0.02-1.4)
Gestational age			
1st trimester	1		
2^nd^-3^rd^ trimester	0.90 (0.46-1.77)	0.760	—
Water source			
Safe	1		1
Unsafe	3.2 (1.6-6.7)	0.002	2.8 (1.3-6.2)
Boiling drinking water			
Yes	1		—
No	4.2 (0.98-17.8)	0.054	
Pit latrine utilization			
Yes	1		1
No	3.9 (1.4-11.0)	0.009	3.2 (1.1-9.8)
Gardening shoes			
Yes	1		1
No	4.3 (2.0-9.2)	<0.001	3.4 (1.6-7.5)
Floor type			
Concrete	1		—
Earth	2.7 (0.80-9.1)	0.108	

^a^OR: odds ratio.

## Data Availability

The data used to support findings of this study are available from the corresponding author upon request.
